# Use of the JAK Inhibitor Ruxolitinib in the Treatment of Hemophagocytic Lymphohistiocytosis

**DOI:** 10.3389/fimmu.2021.614704

**Published:** 2021-02-16

**Authors:** Camille Keenan, Kim E. Nichols, Sabrin Albeituni

**Affiliations:** Department of Oncology, St. Jude Children's Research Hospital, Memphis, TN, United States

**Keywords:** Hemophagocytic lymphohistiocytosis, ruxolitinib, jak-stat, inflammation, cytokine storm

## Abstract

Hemophagocytic lymphohistiocytosis (HLH) is a rare hyperinflammatory syndrome driven by overactive T cells and macrophages that abundantly secrete numerous pro-inflammatory cytokines, including interferon (IFN)-gamma, interleukin (IL)-1-beta, IL-2, IL-6, IL-10, IL-18, and tumor necrosis factor (TNF). The release of these and other cytokines underlies many of the clinical and pathologic manifestations of HLH, which if left untreated, can lead to multi-organ failure and death. The advent of etoposide-based regimens, such as the Histiocyte Society HLH-94 and HLH-2004 protocols, has substantially decreased the mortality associated with HLH. Nevertheless, the 5-year survival remains low at ~60%. To improve upon these results, studies have focused on the use of novel cytokine-directed therapies to dampen inflammation in HLH. Among the agents being tested is ruxolitinib, a potent inhibitor of the Janus Kinase (JAK) and Signal Transducer and Activation of Transcription (STAT) pathway, which functions downstream of many HLH-associated cytokines. Here, we review the basic biology of HLH, including the role of cytokines in disease pathogenesis, and discuss the use of ruxolitinib in the treatment of HLH.

## Introduction

Hemophagocytic lymphohistiocytosis (HLH) is a rare and life-threatening hyperinflammatory syndrome resulting from inherited (primary HLH) or acquired (secondary HLH) immune dysregulation. The excessive immune response is associated with T and myeloid cell infiltration of numerous organs, such as the liver, spleen, and central nervous system, where activated immune cells profusely secrete cytokines and mediate significant tissue damage. If not promptly treated, the exuberant immune response can lead to multiorgan failure and death (1). The diagnosis of HLH is based on criteria established by the Histiocyte Society in 1994 and revised in 2004, and should be strongly suspected in individuals exhibiting features such as fever, splenomegaly, hyperferritinemia, bi- or tri-lineage cytopenias, hypofibrinogenemia or hypertriglyceridemia, elevated soluble CD25, hemophagocytosis, and reduced or absent NK cell cytotoxicity (2). HLH thus constitutes a fascinating syndrome wherein familial or acquired defects in immunity result in hyperinflammation, as opposed to the phenotype of reduced immune function leading to chronic or rare infections as is seen with most other immunodeficiency syndromes (3).

## Epidemiology and Genetics of HLH

Primary HLH is estimated to affect one in every 50,000–100,000 children (4, 5) and is caused by pathogenic germline variants that negatively impact CD8+ T and natural killer (NK) cell cytotoxicity. Primary HLH is autosomal recessive and caused by homozygous or compound heterozygous mutations in the genes *PRF1, UNC13D, STX11*, and *STXBP2*, which are required for the proper function and extrusion of perforin-containing cytolytic granules. Patients with the autosomal recessive syndromes of pigmentary dilution and impaired lysosomal formation or lytic granule secretion such as Chediak-Higashi syndrome (due to pathogenic variants in *LYST*) and Griscelli syndrome (*RAB27A*) are also at risk to develop HLH, as are patients with the X-linked disorders X-linked lymphoproliferative syndrome 1 (*SH2D1A*) and 2 (*XIAP*) (6).

Although its true incidence is not known, secondary HLH occurs in patients without bi-allelic HLH gene variants, a familial pattern of HLH, or evidence of prior immunodeficiency, who develop a hyperinflammatory phenotype following exposure to a strong immunologic trigger. Such triggers include a variety of infections (viruses being most common), malignancies (generally T or NK cell lymphomas), and autoimmune disorders [such as systemic juvenile idiopathic arthritis (SJIA), where the HLH-like manifestations are often referred to as macrophage activation syndrome (MAS)] (7). Rare patients with secondary HLH have been identified to harbor monoallelic pathogenic germline variants in the genes known to be associated with primary HLH; however, it remains questionable whether these variants play a role in the development of disease (8, 9).

## HLH Pathogenesis

Initial insights into the pathogenesis of primary HLH were provided in 1999 with the seminal discovery of the first primary HLH gene, *PRF1*, which encodes the pore forming protein perforin (10). Over the next decade, several additional primary HLH genes were identified, each of which plays an essential role in granule-mediated lymphocyte cytotoxicity. Studies using mouse genetic models have revealed that defects in these genes impair lymphocyte cytotoxicity leading to poor clearance of pathogens and activated antigen presenting cells and resulting in an ever-spiraling feed forward loop of immune cell activation without the ability to end this cycle.

The pathogenesis of secondary HLH is less well understood. One proposed mechanism is that aberrant stimulation of Toll like receptors (TLR) leads to excessive activation and cytokine production by cells of the innate immune system (11). This possibility is intriguing since Epstein Barr virus (EBV), one of the most common infectious triggers of secondary HLH, engages TLR9 to stimulate myeloid cells. Another possible mechanism involves the inflammasome-dependent overproduction of IL-18 by activated myeloid and epithelial cells. Excess free IL-18 amplifies lymphocyte production of IFN-gamma and is proposed to contribute to MAS (12).

Regardless of their underlying differences in pathogenesis, one of the features shared by primary and secondary HLH is the excessive production of pro-inflammatory cytokines with IFN-gamma, TNF, IL-1, IL-2, IL-6, IL-10, IL-12, IL-18 and granulocyte macrophage-colony stimulating factor (GM-CSF) representing some of the cytokines most notably elevated (13–16). Based on these findings, several studies have sought to determine which of these cytokines is most required for the development and/or propagation of HLH. Toward this end, a report by Binder et al. in 1998 (1 year before perforin-deficiency was identified as a cause for primary HLH) described the development of an HLH-like syndrome in perforin-deficient mice following infection with Lymphocytic Choriomeningitis Virus (LCMV) (17). Disease manifestations were similar to those observed in humans with HLH including expansion of activated CD8+ T cells, pancytopenia and excessive production of TNF and IFN-gamma (17). In this report, depletion of CD8+ T cells, genetic ablation of TNF, and/or antibody-mediated neutralization of IFN-gamma, each significantly improved bone marrow function and enhanced survival of LCMV-infected animals. Subsequent studies using perforin or RAB27A-deficient mice have confirmed these initial findings, with depletion of CD8+ T cells or blockade of IFN-gamma (but not TNF, IL-12, IL-18 or GM-CSF) improving survival and lessening the manifestations of disease (18, 19).

While these data point to a central role for IFN-gamma in the pathogenesis of primary HLH, a recent report by Burn et al. reveals that mice lacking expression of both perforin and IFN-gamma develop a severe HLH-like illness following LCMV infection, although the characteristics of this illness differ from those observed in animals lacking expression only of perforin. Specifically, perforin- and IFN-gamma-double deficient mice develop a marked neutrophilia and a serum cytokine pattern notable for increased IL-6, IL-1-beta and GM-CSF but not increased IFN-gamma (20). These data strongly suggest that cytokines in addition to IFN-gamma likely also play important roles in the development and/or progression of HLH-associated hyperinflammation.

## Approaches to HLH Treatment

### Primary HLH

The mainstay of primary HLH treatment involves generalized suppression of the immune system, usually with steroids and chemotherapy. Without such treatment, primary HLH is almost always fatal. Notably, cure can only be obtained via allogeneic hematopoietic stem cell transplantation (HSCT) in which the defective immune system is replaced with a healthy one. Like hematologic malignancies, active HLH at the time of transplant may portend a poorer outcome (21, 22).

The advent of etoposide-based regimens, such as the HLH-94 and HLH-2004 protocols, has substantially increased the survival for patients with newly diagnosed primary HLH (1). Indeed, use of the HLH-94 protocol resulted in a 54% 5-year probability of survival following treatment with etoposide and dexamethasone, delayed cyclosporine, and intrathecal (IT) methotrexate for patients with central nervous system (CNS) involvement, followed by HSCT (21). Nevertheless, 29% of patients died before HSCT and 19% developed late neurologic sequelae. To reduce pre-transplant mortality and neurologic complications, the HLH-2004 protocol added up-front cyclosporine and included IT corticosteroids. Although HLH-2004 resulted in a 5-year estimated survival of 62%, neither treatment modification significantly improved overall outcome (23). Therefore, the HLH-94 regimen remains the current standard of care in most centers.

For patients with relapsed/refractory primary HLH, there are limited data on the utility of specific salvage therapies. Case reports and retrospective case series describe the use of the anti-CD52 antibody alemtuzumab and anti-thymocyte globulin (ATG); however, there are no published prospective trials using these agents. A retrospective study of alemtuzumab revealed that 64% of patients with relapsed/refractory HLH responded and 77% survived to HSCT (24). These less-than-perfect outcomes have prompted investigation of alternative therapeutic strategies. Toward this end, a recent report describes 27 patients with relapsed/refractory primary HLH treated with dexamethasone, the IFN-gamma neutralizing antibody emapalumab, and varying combinations of other agents (25). The overall response for these patients was 63% with 70.4% proceeding to HSCT. In this study, seven patients received emapalumab as first line therapy with none achieving a complete response (CR) despite IFN-gamma being a prominent cytokine in primary HLH.

### Secondary HLH

When possible, the treatment of secondary HLH should always begin by targeting the underlying trigger using agents to combat infection, malignancy or auto-immune disease. However, in some cases it may also be necessary to treat the associated hyperinflammation. Various agents have been used, including dexamethasone and, in the most severe cases, etoposide. Agents that block the effects of individual cytokines have also been employed, with this approach particularly appealing for patients whose HLH is triggered by infection, where the administration of cytotoxic or other immunosuppressive drugs could compromise clearance of the inciting pathogen.

One such study retrospectively examined 44 patients with secondary HLH/MAS who received treatment with recombinant IL-1 receptor antagonist, anakinra. This study revealed a 73% survival with treatment most successful when given within the first 5 days of hospitalization and to those with underlying rheumatologic diseases such as SJIA (26). A current trial is ongoing to assess the efficacy of the recombinant IL-18 binding protein, Tadekinig alfa, in patients with MAS who harbor germline *NLRC4* pathogenic variants or patients with *XIAP* deficiency (NCT03113760). The anti-IL-6R antibody, tocilizumab, has been reported to mitigate the signs and symptoms of cytokine overproduction [a condition known as cytokine release syndrome (CRS)] in cancer patients receiving treatment with chimeric antigen receptor transduced T (CAR-T) cells (27). Since the cytokine patterns in CAR-T-treated patients are similar to those observed in patients with HLH, IL-6 blockade might prove beneficial in the treatment of HLH. Supporting this notion, there is emerging evidence that tocilizumab can induce remission in adults with secondary HLH (28, 29).

## JAK Inhibition in Pre-Clinical Models of HLH

**Figure 1:**
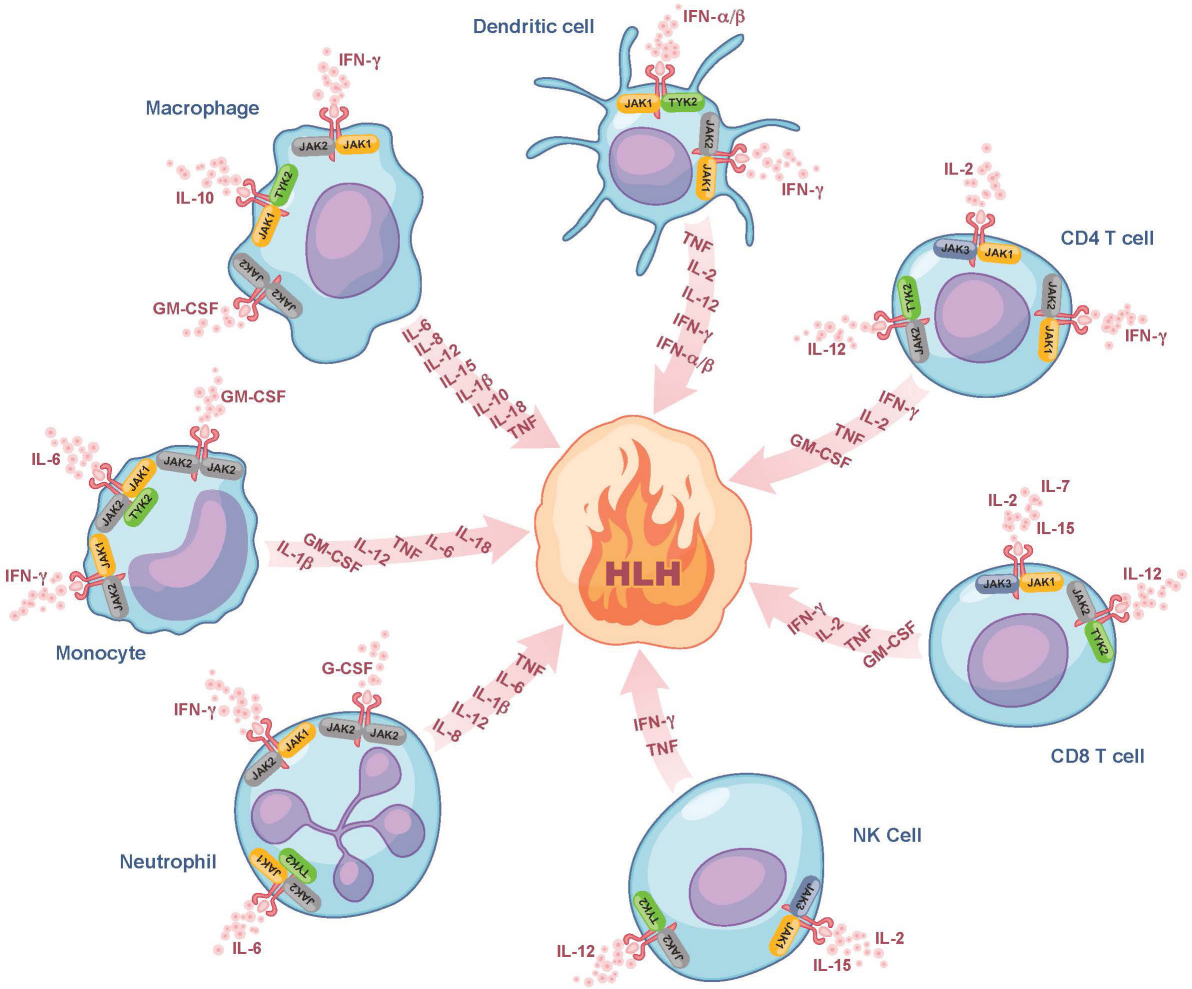
Role of JAK/STAT signaling in fueling the fire of HLH. Here we show several of the cytokines that fuel the fire of HLH and their associated cellular sources. We also illustrate which of these cytokines bind to receptors associated with JAK-family kinases (JAK1, JAK2, JAK3, or TYK2) and are expressed on the surface of dendritic cells, CD4 and CD8 T cells, NK cells, neutrophils, monocytes, and macrophages. These JAK-dependent receptor-mediated signaling pathways represent potential targets of ruxolitinib. Note that many of the cytokines produced are also those that trigger cell activation and hence part of the feedforward loop. For simplicity, all receptors are drawn similarly, but it is noted that there exist some receptors that typically form complexes (e.g., GM-CSF), which are not depicted in this Figure. G-CSF, granulocyte colony-stimulating factor; GM-CSF, granulocyte-macrophage colony-stimulating factor; HLH, hemophagocytic lymphohistiocytosis; IFN-α/β, interferon alpha and beta; IFN-γ, interferon gamma; IL, interleukin; JAK, Janus kinase; NK cell, natural killer cell; TNF, tumor necrosis factor; TYK2, tyrosine-protein kinase 2.

Despite the use of currently available therapies, there are many patients with HLH who succumb to the disease. It is thus noteworthy that many of the cytokines elevated in HLH, such as IFN-gamma, IL-2, IL-6, IL-10, IL-12, and GM-CSF signal through a pathway involving the Janus kinases (JAKs) and Signal Transducers and Activators of Transcription (STATs) (Figure 1) (30). By dampening signaling downstream of numerous HLH-associated cytokines, the interruption of the JAK-STAT pathway holds promise to more effectively lessen HLH-associated immunopathology. Numerous JAK inhibitors, such as ruxolitinib, tofacitinib, baricitinib, and oclacitinib have been used for the treatment of inflammatory disorders (31). In this review, we will focus on ruxolitinib as it is the JAK inhibitor for which there are the most published data on its use in the treatment of patients with HLH.

Ruxolitinib (Jakafi®) is an oral, potent, highly bioavailable JAK1/2 inhibitor that is approved by the Food and Drug Administration (FDA) for use in patients with myeloproliferative neoplasms and steroid refractory graft-vs.-host-disease (GVHD), where it has shown to induce responses, even in those with the most advanced disease (32–34). Currently, ruxolitinib is being trialed as GVHD prophylaxis for patients undergoing allogeneic HSCT (35, 36). In addition, ruxolitinib has been reported to lessen inflammation in patients with pathogenic germline *STAT1* or *STAT3* gain-of-function (GOF) variants. In one patient with a *STAT3* GOF variant, combined treatment with ruxolitinib and tocilizumab led to HLH remission (37). Given the efficacy of ruxolitinib in these other disorders, we and Maschalidi et al. independently examined its activity in mouse models of HLH. Specifically, we used ruxolitinib to treat LCMV-infected perforin- or RAB27A-deficient mice (models of primary HLH), as well wild-type mice exposed to repeated injections of CpG DNA (model of secondary HLH) (11, 38). Through these approaches, we demonstrated that ruxolitinib reversed many HLH manifestations, including splenomegaly, cytopenias, hypercytokinemias, peripheral organ and CNS inflammation, and it significantly prolonged survival (39, 40).

Ruxolitinib inhibits both JAK1 and 2, which function downstream of IFN-gamma as well as several other HLH-associated cytokines, such as IL-2 IL-6, IL-10, IL-12, and GM-CSF. As a consequence, it remained unknown whether the beneficial effects of ruxolitinib resulted simply from its targeting of IFN-gamma, or instead from targeting of other cytokine signaling pathways. To gain further insights, we directly compared the effects resulting from ruxolitinib treatment vs. those obtained following administration of an equipotent dose of IFN-gamma-blocking antibody in the mouse models of HLH. Through this study, we observed that IFN-gamma was the main driver of anemia, but cytokines other than IFN-gamma promoted HLH-associated immunopathology. Supporting this observation, both ruxolitinib and the anti-IFN-gamma blocking antibody improved hemoglobin levels, but only ruxolitinib significantly reduced the number and activation status of immune cells, as well as their infiltration into various tissues (41).

Although single agent ruxolitinib greatly reduces inflammation in mouse pre-clinical models of HLH, disease manifestations are never completely eliminated. Therefore, we most recently examined whether treatment with dexamethasone and ruxolitinib might exert superior control of disease. In this study, we also show that elevated levels of STAT5-dependent cytokines such as IL-2, IL-7 and IL-15 induce resistance of activated CD8+ T cells to glucocorticoid-induced cell death. Notably, by blocking cytokine signaling with ruxolitinib, we could sensitize CD8+ T cells to dexamethasone-induced apoptosis *in vitro* and with combination therapy significantly reducing tissue immunopathology and HLH disease manifestations *in vivo* (42).

## Ruxolitinib Treatment of Human Patients With HLH

Since the initial reports by Das et al. and Maschalidi et al. describing use of ruxolitinib in mouse HLH models, there has been an increasing number of publications describing the use of this drug in patients with HLH. At the time of writing of this review, 18 independent studies describing 115 unique patients have been published, with most studies including adults with secondary HLH for whom ruxolitinib was incorporated as part of a salvage regimen (Table 1). In many cases, ruxolitinib improved clinical manifestations, such as fever, shock, renal failure, and respiratory depression within as little as 24–48 h following initiation of drug. In addition, serum levels of ferritin, soluble IL-2 receptor, and fibrinogen often normalized within seven to 30 days. Overall, ruxolitinib is described as being well-tolerated at doses ranging from 2.5 to 25 mg with the medication administered twice daily. Below, we describe several studies that used ruxolitinib for patients with primary or secondary HLH (focusing here on secondary HLH due to infections or rheumatologic diseases). Malignancy-associated HLH will not be discussed; however, the reader is referred to reports describing ruxolitinib use in this population, as outlined in Table 1.

**Table 1:** Summary of publications describing use of ruxolitinib in patients with HLH.

**Reference**	**N**	**Mean age (range)**	**HLH type**	**Therapy (N)**	**Ruxolitinib dose**	**Ruxolitinib duration**	**Concurrent HLH treatment (N)**	**ORR (CR, PR)**	**Overall survival^†^**
Acosta et al. (43)	1	35 y	Secondary *(EBV/CMV, HIV)*	Frontline	15 mg BID	4 weeks	Dex	100% (CR)	100% (4 weeks)
Ahmed et al. (44)	7	41 y (29–62)	Secondary *(1 AOS, 1 CMV, 1 SLE, 4 unknown)*	Frontline (5) Rel/Ref (2)	15 mg BID	14–28 days	None (2) Steroids (5)	100% (43%, 57%)	100% (60 days)
Broglie et al. (45)	1	11 y	Secondary *(unknown)*	Rel/Ref	2.5 mg BID	NR	Dex & Anakinra	100%	100% (30 days)
Fuchs et al. (46)	1	33 y	Secondary *(Falciparum malaria)*	Rel/Ref	5 mg BID	6 weeks	Dex & Etoposide	100%	100% (3 months)
Goldsmith et al. (47)	2	25 y (24–26)	Secondary *(1 EBV, 1 SJIA)*	Rel/Ref	5–20 mg BID	3 months (1) NR (1)	None (1) Alemtuzumab & steroids (1)	100% (CR)	100% (3 months)
Jianguo et al. (48)	1	6 y	Secondary *(PEG-IFNα-2a for chronic HBV)*	Frontline	2.5 mg BID	3 months	None	100% (CR)	100% (1 year)
Levy et al. (49)	1	16 y	Secondary *(SPTCL, TIM3 def.)*	Rel/Ref	15 mg BID 20 mg BID	2 months 10 months	Cyclosporine & Anakinra	100% (CR)	100% (19 months)
Ramanan et al. (50)	1	3 y	Primary *(UNC13D)*	Rel/Ref	2.5 mg BID	4 weeks	Steroids	100% (CR)	100% (4 weeks)
Sin et al. (51)	1	38 y	Secondary *(EBV)*	Rel/Ref	20 mg BID (10 mg BID w/ renal failure)	7 days	None	100% (PR)	0% (7 days)
Slostad et al. (52)	1	71 y	Secondary *(Histoplasmosis, RA)*	Frontline	10 mg BID	8 days +3 wk taper	Steroids	100%	100% (3 weeks)
Trantham et al. (53)	2	45 y (24–66)	Secondary *(lymphoma)*	Rel/Ref	10–15 mg BID	NR	None (1) Alemtuzumab & anakinra (1)	100% (CR1^§^)	0% (8 months)
J Wang et al. (54)	34	25.5 y (2–70)	Primary *(1 PRF1)* Secondary *(25 EBV, 2 MAS, 6 unknown)*	Rel/Ref	0.3 mg/kg/day 5 mg BID (<14 y, ≥25 kg) 2.5 mg BID (<25 kg)	NR	None (16) Steroids (18)	73.5% (14.7% 58.8%)	56% (4–52 weeks)
H Wang et al. (55)	3	40 y (24–52)	Secondary *(2 lymphoma, 1 AOS)*	Frontline	5 mg BID	4 weeks +1 wk QD	Dex & Etoposide	100% (33% CR, 66% PR)	33%
Wei et al. (56)	9	1.7 y (9m−5y)	Primary *(2 PRF1)* Secondary *(5 EBV, 1 autoinflammatory disease, 1 unknown)*	Rel/Ref	2.5 mg BID (<10 kg) 5 mg BID (10–25 kg) 10 mg BID (>25 kg)	1–4 weeks	NR	33% (33% CR, 0% PR)	89% (10–15 months^*^)
Zandvakili et al. (57)	1	72 y	Secondary *(Histoplasmosis, IBD, RA)*	Frontline	10 mg BID	5 days +3 wk taper	None	100% (CR)	100% (6 weeks)
Zhang et al. (58)	12	4.7 y (1–13)	Secondary *(8 EBV, 2 autoinflammatory disorder, 2 unknown)*	Frontline	2.5 mg BID (≤10 kg) 5 mg BID (11–20 kg) 10 mg BID (>20 kg)	3–28 days	None	83.3% (66.7% CR, 8.3% PR)	91.7% (10 months^**^)
Zhao et al. (59)	1	14 y	Primary *(RAB27A)*	Rel/Ref	5–25 mg BID	2 months	Steroids	100%	100% (8 months)
Zhou et al. (60)	36	44.7 y (31–58)	Secondary *(lymphoma)*	Frontline	0.3 mg/kg/day	14 days	Dex, Etoposide, Doxorubicin	83.3% (27.8% CR, 55.6% PR)	40% (5 months)

*AOS, Adult-onset Stills disease; BID, twice daily; CMV, Cytomegalovirus; CR, complete response; Dex, Dexamethasone; EBV, Epstein-Barr virus; HBV, Hepatitis B virus; IBD, Inflammatory bowel disease; NR, Not reported; PR, partial response; QD, once daily; RA, Rheumatoid arthritis; Rel/Ref, Relapsed/Refractory; SJIA, Systemic juvenile idiopathic arthritis; SLE, Systemic lupus erythematosus; SPTCL, Subcutaneous panniculitis-like T-cell lymphoma*.

† *Overall survival reported as percentage patients alive at publication, with time from initiation of ruxolitinib to end point (last follow-up or death) in parentheses when available*.

* *Survival reported only for patients who completed full 4 weeks ruxolitinib since therapy was switched if no CR by 1 week*.

** *Survival reported only for patients who received ruxolitinib alone and did not go on to receive HLH-94 therapy*.

§ *Both patients achieved CR1, but both subsequently relapsed (one while on ruxolitinib taper, one after 24 days ruxolitinib)*.

### Salvage Therapy

The first report describing use of ruxolitinib as a treatment for HLH included an 11-year old boy with refractory disease and no identifiable trigger (45). Despite treatment with etoposide and dexamethasone, his HLH did not go into remission and his status deteriorated. The subsequent addition of ruxolitinib induced a rapid clinical response and within 24 h he had no fever and exhibited improved respiratory and liver function. The levels of ferritin and soluble IL-2 dropped within a month and the patient ultimately underwent a successful allogeneic HSCT.

Several additional studies have further described favorable responses to ruxolitinib when given as a component of salvage therapy. For example, Goldsmith et al. reported that treatment with ruxolitinib in conjunction with alemtuzumab induced remission in a patient with SJIA whose HLH/MAS failed to respond to treatment with HLH-94 therapy (47). One report from China describing use of ruxolitinib and corticosteroids to treat 34 children and adults with secondary HLH revealed a response rate of 74%, with 14.7% complete responses (CR) and 58.8% partial responses (PR), and 56% overall survival (54). Within 24 h of starting ruxolitinib, 30 patients (88%) became afebrile and ferritin and soluble CD25 levels were significantly lower after 2 weeks. In this study, complete or partial responses correlated with reduced levels of serum cytokines such as IFN-gamma, IL-18, Macrophage Inflammatory Protein (MIP)-1-alpha, and IFN-gamma-Inducible Protein (IP)-10. Several patients in this study relapsed, most notably those with EBV-related HLH, suggesting that early allogeneic HSCT is required for this population despite an initial positive clinical response to ruxolitinib (55). Ruxolitinib has been used in six pediatric patients with primary HLH whose disease relapsed before or after allogeneic HSCT, with all six patients exhibiting a complete or partial response (50, 56, 59). Overall, these and other studies suggest that ruxolitinib can effectively dampen inflammation in this challenging patient population. Nevertheless, as many patients ultimately relapse, allogeneic HSCT should still be considered the ultimate curative therapy for those who are able to tolerate the procedure. Given the profound immune suppression that accompanies repeated courses of therapy, patients require careful monitoring for and prophylaxis against infection.

### Front-Line Therapy

Ruxolitinib has been trialed as a first-line therapy for HLH, albeit to a lesser extent than as a salvage therapy. This was first reported by Slostad et al. (52) and Zandvakili et al. (57) who each described a single adult patient with secondary HLH presenting in the context of histoplasma infection. Given their older age and critical illness, including worsening cytopenias, septic shock, renal, hepatic, and respiratory failure, ruxolitinib was given as a first-line therapy along with anti-fungal agents. For both patients, fever, multi-organ failure and cytopenias improved with full recovery and normalization of HLH-related laboratory parameters within 6 weeks.

More recently, Ahmed et al. reported the results of an open-label, prospective, single-center study enrolling five adults with newly diagnosed HLH (44). Two additional patients were included in the report despite being previously treated. Six of these patients had secondary HLH and one with systemic lupus erythematosus was later found to harbor a homozygous germline *STXBP2* mutation. In all treated patients, cytopenias and serum ferritin levels improved within the first one to 2 weeks of treatment. Notably, the 2-month overall survival was 100% with no deaths reported after a median follow up of 490 days. Adverse events possibly related to ruxolitinib included one patient with Grade 4 neutropenia, and single patients each with Grade 1 or 2 skin findings (maculopapular rash, intermittent candidiasis), nausea or extremity pain.

Extending these studies to children, Zhang et al. used ruxolitinib to treat 12 pediatric patients with newly diagnosed secondary HLH, including eight with EBV-triggered disease (58). Ten patients (83%) experienced a favorable response at 28 days, with eight exhibiting a CR. Among these eight, seven (88%) maintained the CR for more than 6 months. The remaining four patients did not respond or deteriorated after ruxolitinib treatment, requiring subsequent therapy according to the HLH-94 protocol. Together, these studies suggest that ruxolitinib may prove useful for the initial treatment of secondary HLH. Nevertheless, the optimal dose and schedule of ruxolitinib administration remain to be determined. In addition, studies examining regimens including ruxolitinib and other HLH-directed agents for the treatment of infants and young children with primary HLH are needed to better understand the utility and safety of ruxolitinib in these settings.

## Concluding Remarks and Future Directions

The treatment of primary HLH has advanced tremendously over the last 30 years from a disease that was uniformly fatal to one where the majority of patients can now be cured. Nevertheless, there are still many patients with HLH who succumb to the disease or to the complications of its treatment. Therefore, novel approaches to therapy are desperately needed. Thanks to increased understanding of the roles played by cytokines in fueling the fire of HLH, studies of cytokine-targeting agents such as ruxolitinib have been initiated with emerging data suggesting that some of these agents, including ruxolitinib, represent potentially effective means to lessen inflammation in this disease. However, it is not yet possible to discern the true efficacy of ruxolitinib for children or adults with HLH without prospective clinical trials, preferably using standardized definitions of disease activity and response and comparing with current gold-standard therapies.

The cytokine patterns observed in HLH closely resemble those seen in other hyperinflammatory conditions, such as the CRS that commonly follows treatment with T cell-based cancer immunotherapies (61). Notably, treatment with ruxolitinib in a pre-clinical xenograft model prevented the onset of CRS after administration of CD123-directed CAR-T cells and it did so without affecting the anti-tumor activity of the CAR-T cells (62). Therefore, it is possible that ruxolitinib or other JAK inhibitors could prove beneficial in ameliorating the signs and symptoms of immunotherapy-associated CRS.

Similarly, a cytokine storm has been observed in patients infected by the novel coronavirus SARS-CoV-2, where there is upregulation of cytokines including IL-1 and IL-6 (63). Accordingly, anakinra, tocilizumab, and ruxolitinib are being trialed for this disease (64, 65). Of late, studies have reported that treatment of COVID-19 patients with baricitinib, a structural analog of ruxolitinib, reduces serum cytokine levels, restores lymphocyte counts, increases the levels of antibodies reactive against the SARS-CoV2 spike protein, and lessens viral burden (66). In line with these findings, patients with COVID-19 and moderate pneumonia who receive treatment with baricitinib exhibit improved clinical status, reduced need for supportive care and ICU admission, and decreased recovery time (66–68). Based on these data, baricitinib was granted emergency use authorization by the FDA to be given in combination with the anti-viral agent remdesivir for the treatment of suspected or laboratory confirmed COVID-19 in hospitalized adults and pediatric patients 2 years of age or older who require supplemental oxygen, invasive mechanical ventilation, or extracorporeal membrane oxygenation (69). All told, the future is looking brighter for patients with HLH and related disorders of the immune system thanks to the availability of ruxolitinib and other inhibitors of the JAK-STAT pathway.

## Author Contributions

CK and SA wrote the manuscript. KN: reviewed and edited the manuscript. All authors contributed to the article and approved the submitted version.

## Conflict of Interest

KN receives research funding from Incyte Pharmaceuticals. The remaining authors declare that the research was conducted in the absence of any commercial or financial relationships that could be construed as a potential conflict of interest.
